# Balancing act of cytokines in controlling the severity of *Clostridioides difficile* infection

**DOI:** 10.1128/iai.00551-25

**Published:** 2026-07-17

**Authors:** Kevin S. Mears, Michael C. Abt

**Affiliations:** 1 Department of Microbiology, Perelman School of Medicine, University of Pennsylvania 332252, Philadelphia, Pennsylvania, USA; 2 Institute for Immunology and Immune Health, Perelman School of Medicine, University of Pennsylvania 6572, Philadelphia, Pennsylvania, USA; University of California at Santa Cruz, Santa Cruz, California, USA

**Keywords:** *Clostridium difficile*, cytokines

## Abstract

The severity of *Clostridioides difficile* infection is dictated by a complex interplay of various host cell types and inflammatory mediators responding to the pathogen and surrounding microbiome. An effective immune response must carefully balance controlling *C. difficile*-mediated pathology and systemic dissemination of opportunistic bacteria while avoiding collateral immunopathology. In addition, a successful immune response must also promote restorative mechanisms to repair toxin-induced disruptions to the intestinal barrier. Here, we review the immune response to *C. difficile* infection with a specific focus on the role of innate immune-induced cytokines.

## *C. DIFFICILE* INFECTION IS A CAUSE OF SIGNIFICANT MORBIDITY

*Clostridioides difficile* is the most common cause of health care-associated gastrointestinal infections and is responsible for approximately half a million infections and 30,000 deaths in the United States annually (1–3). *C. difficile* colonizes the large intestine and causes a range of symptoms from mild diarrhea to pseudomembranous colitis, toxic megacolon, and sepsis, which may lead to death (4). The most significant risk factor for *C. difficile* infection is antibiotic use, particularly broad-spectrum antibiotics (5, 6). Additional risk factors for *C. difficile* infection include advanced age (65 or older), weakened immune system, and co-morbidities such as inflammatory bowel disease (IBD) (6). Despite recent declines in health care-associated cases of *C. difficile* infection, incidences of community-associated *C. difficile* infection, which is responsible for approximately one-third of total *C. difficile* infection cases, have remained steady after adjusting for the introduction of more sensitive diagnostic tests (2).

## PATHOGENESIS OF *C. DIFFICILE* INFECTION

Following ingestion of spores and germination into actively dividing vegetative cells, *C. difficile* rapidly colonizes the large intestine and initiates the production of two primary virulence factors, *C. difficile* toxin A (TcdA) and *C. difficile* toxin B (TcdB). These secreted toxins are important for driving inflammation and causing disease (7). The toxins contain a glucosyltransferase domain (GTD) responsible for the cytopathic effects of TcdA and TcdB (8). The GTD catalyzes the monoglucosylation of members of the Rho family of GTPases, including RhoA, Rac1, and Cdc42, leading to their inactivation (9, 10). Inactivation of Rho-family GTPases leads to morphological changes in intestinal epithelial cells, disruption of epithelial tight junctions, and cell death (8). This further leads to the release of proinflammatory cytokines and chemokines that initiate the acute inflammatory response, characterized by robust neutrophil recruitment to the large intestine, which can present as raised plaques in patients with pseudomembranous colitis (11, 12).

## HOST SENSING OF *C. DIFFICILE* INFECTION

**Fig 1:**
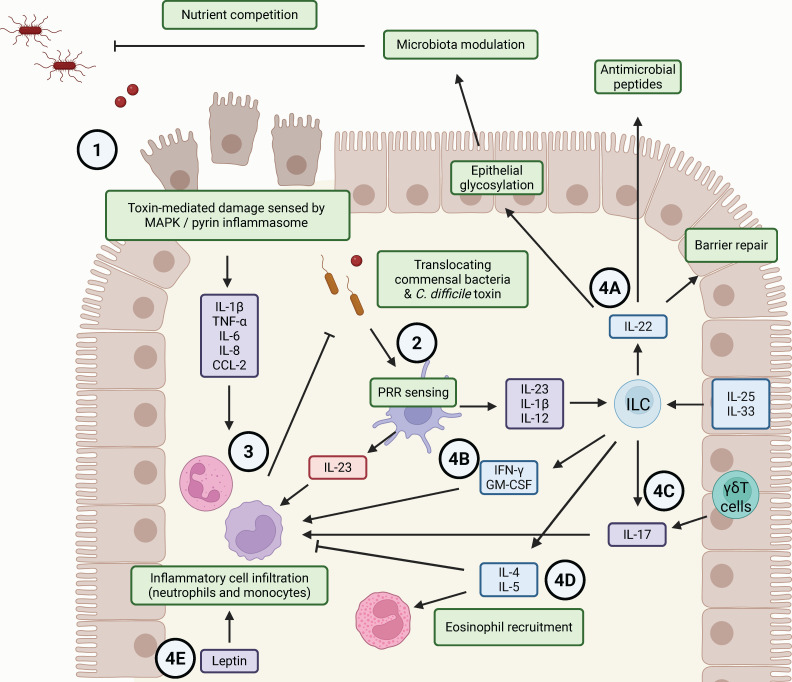
Induction of innate cytokine response following *C. difficile* infection. Red boxes indicate a deleterious impact; blue boxes indicate a beneficial response; purple boxes indicate a combination. (1) Cellular stress induced by *C. difficile* toxins A and B activity is sensed by host MAPK and pyrin inflammasome pathways leading to the release of proinflammatory cytokines and chemokines. (2) PRR sensing of bacterial PAMPs and *C. difficile* toxins leads to the production of cytokines including IL-23, IL-1β, and IL-12. (3) Inflammatory cells, most notably neutrophils and monocytes, are recruited to the site of infection to eliminate translocating commensal microbes. (4A–4E) Following initial innate sensing and cytokine/chemokine response to toxin damage, the innate immune system coordinates a series of cytokine signaling events driven by distinct innate immune cell subsets. (4A) IL-22 produced by ILC3s following IL-23 and IL-1β stimulation signals on the intestinal epithelium and leads to the production of antimicrobial peptides (such as Reg3γ), stimulation of epithelial barrier repair pathways, and induction of epithelial glycosylation that promotes the growth of commensal bacteria, which outcompete *C. difficile* for critical nutrients. (4B) GM-CSF produced by ILC3s and IFN-γ produced by ILC1s further drive the expansion, recruitment, and activation of neutrophils. (4C) IL-17a produced by ILC3s and γδ T cells drive granulopoiesis and neutrophil chemoattraction. (4D) IL-25 and IL-33 stimulate the release of IL-4 and IL-5 from ILC2s, stimulating eosinophil recruitment and suppressing inflammatory responses. (4E) The adipocytokine leptin enhances inflammatory cell recruitment.

TcdA/B-mediated damage of the epithelial barrier and translocation of luminal opportunistic bacteria initiate the acute inflammatory response in the large intestine. Damaged epithelial cells and intestinal-resident immune cells release proinflammatory cytokines and chemokines that recruit and activate antimicrobial activity (13–15)(Figure 1). While this inflammatory response is critical for controlling intestinal infection, overly robust inflammation can lead to increased epithelial damage (16). Indeed, in *C. difficile*-infected patients, the levels of fecal inflammatory markers, such as interleukin (IL)-8, IL-1β, and lactoferrin, and systemic cytokines like IL-6 and TNF-α are better predictors of disease severity than *C. difficile* burden (17–20). These data suggest that fecal samples could be submitted to hospital diagnostic labs to concurrently verify a patient’s *C. difficile* infection diagnosis, along with cytokine readouts to determine the severity of disease to help inform clinicians in the proper course of treatment. During the course of *C. difficile* infection, numerous cytokines are produced to control the infection and facilitate restoration of the damaged intestinal barrier. In the following sections, we discuss the initiation of the acute inflammatory response following infection, the prominent innate cytokines during this response, and their contribution to disease outcome (Table 1).

**TABLE 1:** Cytokines that affect disease severity of *C. difficile* infection

Cytokine	Effect
IFN-γ	IFN-γ producing ILC1s protect against severe *C. difficile* infection (21). *Ifng^−/−^* mice have minimal epithelial damage and inflammation in an ileal loop model of toxin A-induced enteritis (22). Anti-IFN-γ antibody blockade reduced expression of *Cxcl9* and *Cxcl10*, but not monocyte and neutrophil recruitment following *C. difficile* infection (23).
IL-23	Elevated IL-23 in human colon biopsies and stool samples from *C. difficile*-infected patients (24, 25). IL-23 deficient (*Il23p19^−/−^*) mice have reduced disease with attenuated neutrophil recruitment following *C. difficile* infection (24, 26).
IL-22	IL-22 protects against *C. difficile* challenge by regulating complement C3 expression and systemic bacterial clearance (27). Human microbiota-induced IL-22 regulates host epithelial glycosylation to promote commensal nutrient competition (28). Mice lacking the negative receptor, IL-22BP (*Il22ra2^−/−^*), have enriched acetate-producing bacteria that inhibit *C. difficile* colonization (29). TLR7-induced IL-22 from ILC3s facilitates barrier repair processes following infection (30).
IL-17	Elevated Th17 response in *C. difficile-*infected patients with worsened disease outcomes (21). *Il17a* and *Il17f* double knockout mice have reduced disease from *C. difficile* infection with reduced neutrophil infiltration (31). IL-17A-producing γδ T cells are protective against severe disease (27).
IL-4	rIL-4 enhances recovery following *C. difficile* infection (32).
IL-5	rIL-5 reduces *C. difficile* infection severity by increasing eosinophils and reducing neutrophil infiltration (32).
IL-13	rIL-13 reduces *C. difficile* infection severity during the late phase of infection (33).
IL-25	IL-25-induced eosinophilia protects against severe *C. difficile* infection (34). Microbiota-derived succinate increases IL-25-producing tuft cells that protect against severe *C. difficile* infection (35).
IL-33	rIL-33 increases eosinophil infiltration and reduces epithelial damage from *C. difficile* infection (36).

TcdA and TcdB enter a cell through receptor-mediated endocytosis and have different cellular tropisms based on their receptor-binding capacity. To date, both TcdB and TcdA have been found to bind an array of cell surface glycans (37). Additionally, the cell surface proteins chondroitin sulfate proteoglycan 4, Nectin-3, CD44, Frizzled 1 (FZD1), FZD2, and FZD7 have been identified as receptors for TcdB (38–41). The molecular processes of toxin cellular entry and subsequent intoxication have been thoroughly reviewed in the following reports (8, 42, 43). A key initiating step in the innate immune sensing pathway is the release of the toxin’s glucosyltransferase domain into the host cell’s cytosol and subsequent enzymatic transfer of a glucose residue to Rho family proteins, which inactivate the GTPase cycling capacity. GTPase inactivation leads to actin cytoskeleton disruption and cellular stress that are sensed within the intoxicated cell, resulting in the activation of mitogen-activated protein kinases (MAPK) and subsequent expression of proinflammatory factors, including IL-1β, IL-8, TNF-α, and IL-6 (44–49). Intoxication of intestinal epithelial cells leads to the activation of multiple MAPK pathways, including p38, c-Jun N-terminal kinase (JNK), and extracellular signal-regulated protein kinase (Erk) 1/2, and activation of downstream transcription factors NF-κB and AP-1 (13). In particular, p38 MAPK is essential for IL-8 production upon toxin exposure, which drives chemotaxis of neutrophils to damaged intestinal mucosa (44, 50). The p38 MAPK pathway senses disruptions to the actin filaments and mechanical stress (51, 52). Toxin-induced disruption to the cytoskeleton in enterocytes and monocytes causes p38 phosphorylation, thereby triggering p38 kinase-dependent activation of MAPK-activated protein kinase 2 and leading to the increased expression of IL-8, TNF-α, and other inflammatory cytokines (44, 53, 54). Additionally, TcdA, but not TcdB, induces cyclooxygenase (COX)-2 expression in colonocytes through p38 MAPK, leading to the release of prostaglandin E2_2_, a potent stimulator of intestinal water secretion in the intestine (55). Cytoskeletal perturbations from toxin activity also activate JNK and Erk1/2 pathways in intestinal epithelial cells, leading to the formation of AP-1 composed of c-Jun/c-Fos heterodimers and expression of IL-8 (56). Concurrently, modifications to the actin cytoskeleton from TcdB inactivation of Rho GTPase are sensed in monocytes, macrophages, and dendritic cells by the pyrin inflammasome, leading to the formation of an inflammasome that contains the ASC protein (apoptosis-associated speck-like protein containing a caspase recruitment domain) and further caspase-1 activation and release of IL-1β (47). ASC-deficient mice are impaired in neutrophil recruitment that corresponds with reduced production of IL-1β and CXCL1, a functional homolog of human IL-8, and are highly susceptible to *C. difficile* infection (57). *C. difficile* pathogen-associated molecular patterns (PAMPs) are also recognized by host pattern recognition receptors (PRRs) and contribute to the immune response. *C. difficile* surface layer proteins activate toll-like receptor 4 (TLR4) on dendritic cells, leading to their release of IL-12, IL-23, TNF-α, and IL-10 (58). Additionally, *C. difficile* flagellin activates toll-like receptor 5 (TLR5) on intestinal epithelial cells, leading to their release of chemokines IL-8 and CCL20 that recruit immune cells to the site of infection (59).

## INITIATORS OF EARLY INTESTINAL INFLAMMATORY RESPONSE

Following *C. difficile* infection, the innate immune response produces cytokines to coordinate defenses aimed at limiting bacterial dissemination and facilitating restoration of the damaged intestinal barrier. Toxin-induced release of IL-1β and TNF-α stimulates epithelial cells to produce neutrophil-attracting chemokines such as IL-8 and macrophage inflammatory protein-2 (similar to CXCL1, CXCL2, and CXCL5 in mice) and monocyte-attractants like CCL2 that recruit these circulating innate immune cells to the colonic mucosa (60–62). Pseudomembranous colitis presents as yellowish plaques on the surface of colonic mucosa in severe cases of *C. difficile* infection resulting from excessive neutrophil infiltration into the colonic lamina propria and sloughing into the intestinal lumen (63). Excessive neutrophil infiltration has been linked to more severe *C. difficile* disease in the animal model as well (64, 65). Inhibiting granulocyte-macrophage colony-stimulating factor (GM-CSF) and leptin-STAT3 pathways or inducing the prostaglandin E2 pathway reduces neutrophil infiltration and reduces intestinal tissue pathology following *C. difficile* infection (66–68). However, these cells are crucial for the acute protective response following infection, as mice lacking the toll-like receptor (TLR) adaptor molecule MyD88, the ASC inflammasome, or the intracellular bacterial peptidoglycan receptor NOD1 are impaired in CXCL1 production and neutrophil recruitment and are more susceptible to *C. difficile* infection (57, 69, 70). Antibody-mediated depletion of neutrophils also renders mice significantly more susceptible to *C. difficile* infection (70). Additionally, neutropenia is a marker of susceptibility to *C. difficile* in hospitalized leukemia patients (71). Following recruitment to the colonic lamina propria, neutrophils can be activated by toxin to increase production of reactive oxygen species, reactive nitrogen species, and antimicrobial peptides that limit bacterial dissemination from toxin-induced barrier damage (11). While important for acute host defense during infection, chronic activation of neutrophils can lead to collateral tissue damage and exacerbate disease (72). A precise time window post-infection when neutrophils switch from protective to pathogenic is not known and is likely to vary depending on additional contributing factors to pathogenesis (i.e., strain of *C. difficile*, microbiome composition, age, and pre-existing health status of the patient). Most plausibly, successful transition from neutrophil-mediated tissue damage to tissue repair processes following infection is, in part, dictated by local cytokine and chemokine signaling cues that are described in the following section. In addition to the timing of neutrophil recruitment dictating the beneficial or detrimental contribution of this cell type to disease outcome, recent work has started to suggest that subsets within the neutrophil population may have distinct roles. Jose et al. (73) found olfactomedin-4 expressing neutrophils aggregated around epithelial damage and exacerbated toxin-mediated damage. Thus, neutrophils appear to exhibit both protective and pathogenic roles in *C. difficile* disease, which may be able to be explained by parsing out the timing and heterogeneity of neutrophil involvement in the host response to infection.

Inflammasome activation and subsequent IL-1β-IL-1R signaling on myeloid cells, in particular dendritic cells, drive substantial release of interleukin 23 (IL-23) following TLR stimulation or *C. difficile* toxin detection (74, 75). Human colon biopsy specimens positive for *C. difficile* toxins have elevated levels of IL-23 compared to toxin-negative samples (24), and *C. difficile* infection-positive diarrheal stools have greater levels of IL-23 than *C. difficile* infection-negative stools (25). Furthermore, mice lacking IL-23 signaling (*Il23p19^−/−^*) or mice treated with anti-IL-23p19 monoclonal antibody had significantly reduced disease severity and improved survival, indicating the dominant role IL-23 signaling plays in the pathogenesis of *C. difficile* infection (24). IL-23 deficiency in mice leads to reduced levels of neutrophil chemoattractants *Cxcl1*, *Cxcl2*, and *Ccl3* and significant impairment in the recruitment of neutrophils to the colon (26). These defects were independent of IL-22 and IL-17A signaling, indicating that IL-23 has a direct role in neutrophil recruitment during *C. difficile* infection (23, 26). Furthermore, a recent retrospective study found that hospitalized patients with *C. difficile* infection who received anti-IL-23 treatment had significantly improved survival compared to patients who did not receive anti-IL-23 (76). Notably, elevated IL-23 is a key driver of inflammatory bowel disease (IBD) (77), a co-morbidity that is associated with more severe disease in *C. difficile-*infected patients (78–80).

## IFN-γ IS CRITICAL IN ACUTE DEFENSE AGAINST *C. DIFFICILE* INFECTION

In *C. difficile*-infected patients, high levels of IFN-γ are associated with less severe disease (20). The involvement of IFN-γ in *C. difficile* toxin-induced enteritis was first explored by Ishida et al., who showed that IFN-γ expression was elevated following toxin-A treatment in an ileal loop model of toxin-induced enteritis and that IFN-γ was important in the inflammatory response that followed by regulating the expression of TNF-α and CXCL1 (69). Numerous studies have corroborated the induction of IFN-γ expression in mouse models of *C. difficile* infection (31, 66, 81). Furthermore, it was shown that IFN-γ is critical in the protection against acute *C. difficile* infection and is specifically produced by T-bet expressing type 1 innate lymphoid cells (ILC1s) (21). Neutralization of IFN-γ following *C. difficile* infection had no effect on inflammatory monocyte and neutrophil recruitment (23), indicating that IFN-γ protection is downstream of myeloid cell recruitment to the intestine.

## IL-22 PROTECTS AGAINST *C. DIFFICILE* INFECTION THROUGH SYSTEMIC AND LOCALIZED MECHANISMS

IL-22 is also produced early following *C. difficile* infection, and several mechanisms have been proposed for the protective role of IL-22 in *C. difficile* infection. Hasegawa et al. (27) first showed that IL-22-deficient mice (*Il22*^−/−^) are acutely susceptible to a sublethal challenge with *C. difficile*. Mechanistically, they demonstrated that this was independent of inflammatory cell recruitment to the intestine, but instead IL-22 mediated increased complement C3 expression and enhanced clearance of disseminating opportunistic bacteria from peripheral organs by phagocytosis (27). IL-22 also regulates the glycosylation of epithelial mucus (82, 83). Nagao-Kitamoto et al. (28) demonstrated that IL-22 induced by colonization of germ-free *Rag1*^−/−^ mice with a human sourced microbiome protected against intestinal damage following *C. difficile* infection. This protection was driven by IL-22 regulation of host glycosylation, specifically through the upregulation of *Mgat4a* and *Mgat4b*, that enabled the growth of commensal *Phascolarctobacterium* spp. that outcompete *C. difficile* for succinate, thereby limiting *C. difficile* growth (28). A third study explored the role of the soluble decoy receptor IL-22BP, a negative regulator of IL-22 signaling, in the response to *C. difficile* infection. Through co-housing and microbiome transplantation experiments, they found that mice lacking IL-22BP (*Il22ra2*^−/−^) have a microbiome that is enriched for acetate production, which protects against *C. difficile* colonization (29). A recent paper from our group showed that IL-22 from type 3 innate lymphoid cells (ILC3s), induced upon treatment with a TLR7 agonist, protected mice against *C. difficile* infection by facilitating epithelial cell proliferation and survival via epithelial STAT3 signaling (30). This, in turn, reduced toxin-induced mucosal damage and barrier permeability, thereby limiting the systemic dissemination of *C. difficile* toxin (30).

Multiple innate cytokines can contribute to the regulation of IL-22 expression. The primary inducer of IL-22 production is the previously discussed IL-23 (84, 85). IL-1β, produced by various cell types in response to pathogen or cell damage recognition by pattern recognition receptors (PRRs) and downstream inflammasome activation, is also an independent inducer of IL-22 production and can act synergistically with IL-23 (86, 87). In the context of *C. difficile* infection, neutrophil-derived IL-1β can enhance IL-22 production by ILC3s (88).

## IL-17 POTENTIATES THE INFLAMMATORY RESPONSE TO *C. DIFFICILE* INFECTION

The human IL-17 family is comprised of six members: IL-17A (often simply referred to as IL-17), IL-17B, IL-17C, IL-17D, IL-17E (also known as IL-25), and IL-17F, which shares the highest sequence homology to IL-17A (89). Among these, IL-17A is the most extensively studied for its role in inflammatory responses. Similar to IL-23, IL-17A levels are often elevated in the inflamed intestinal mucosa of patients with IBD (90, 91), who are at increased risk for *C. difficile* infection and more severe disease (79, 92). Nakagawa et al. (93) demonstrated that IL-17A/F double knockout mice were more resistant to *C. difficile* infection colitis with a reduction in the levels of inflammatory mediators, IL-1β, granulocyte colony-stimulating factor (G-CSF), CXCL2, and IL-6, and a corresponding reduction in neutrophil accumulation. Saleh et al. (94) used a dextran sodium sulfate (DSS) induced-colitis mouse model, commonly used to model human ulcerative colitis, to show that inflammation driven by an influx of activated CD4^+^ T helper 17 (Th17) cells worsens *C. difficile* infection disease outcome. Furthermore, a shift from Th1 to Th17 CD4^+^ T cell responses was reported in *C. difficile* infection patients with more severe disease compared to those with mild-to-moderate *C. difficile* infection (94). On the other hand, IL-17A produced by γδ T cells has a protective role in *C. difficile* infection and was postulated to be a potential mechanism for the natural immunity infants exhibit against *C. difficile* infection-associated pathologies (95). The contradicting functions of IL-17A in these studies highlight the need to further study the context-dependent role this cytokine plays in the immune response to *C. difficile* infection.

## GM-CSF CONTRIBUTES TO ACUTE DEFENSE AGAINST *C. DIFFICILE* INFECTION

*C. difficile* infection leads to increased intestinal expression of granulocyte-macrophage colony-stimulating factor (GM-CSF) (31), a potent driver of inflammatory cell recruitment (96). Although treating mice with a GM-CSF-blocking antibody had no significant effect on *C. difficile* burden or *C. difficile* infection disease severity, GM-CSF blockade led to reduced expression of inflammatory cytokines IL-1β and TNF-α, neutrophil-recruiting chemokines CXCL1 and CXCL2, and inducible nitric oxide synthase following infection (66). A recent study by Fachi et al. (97) further showed that GM-CSF specifically produced by ILC3s is important for neutrophil infiltration and maturation during *C. difficile* infection, implicating this cytokine as part of the cytokine network coordinating the innate response to infection.

## TYPE 2 CYTOKINES TIP THE BALANCE TOWARD PROTECTIVE EOSINOPHILIC RESPONSE

The type 2 immune response is commonly associated with helminth infections and allergic responses. Despite this, several studies have revealed a protective role for type 2 immunity during *C. difficile* infection. Eosinophils protect the intestinal barrier during *C. difficile* infection (34, 36) (28, 29). In fact, in patients with *C. difficile* infection, an undetectable eosinophil count was associated with worse disease outcomes (98). Approximately 20% of *C. difficile* strains, including the epidemic strain NAP1/027, encode a third toxin, binary toxin (CDT) (99, 100). CDT suppresses protective eosinophil responses via a TLR2-dependent pathway (101). Elevated levels of systemic IL-5, which support eosinophil recruitment, are associated with reduced disease severity in *C. difficile*-infected patients (20). In mice, treatment with recombinant IL-5 shifts the balance of neutrophils and eosinophils infiltrating the colonic lamina propria following infection to reduce the severity of *C. difficile* infection (32). Recombinant IL-4 and IL-13 treatments are also protective but only by enhancing recovery during the later phase of infection (32, 33). IL-25, produced by tuft cells in the intestines, is an inducer of IL-4, IL-5, and IL-13. Buonomo et al. (34) demonstrated that IL-25 levels are also reduced in both *C. difficile*-infected patients and following infection in mice. Furthermore, pretreatment of mice with recombinant IL-25 protects against severe disease without affecting pathogen burden or toxin levels in the intestine. This IL-25 treatment increased eosinophil accumulation in the colonic lamina propria, which was necessary for protection of the epithelial barrier (34). A recent study further demonstrated that a microbiome enriched in succinate-producing bacteria increases colonic tuft cells and IL-25 to protect against severe *C. difficile* infection (35). IL-33, an alarmin released by damaged epithelial cells, is another inducer of type 2 immunity leading to the production of IL-4, IL-5, and IL-13 from a variety of cells including Th2 cells and ILC2s (102). *C. difficile* infection leads to an upregulation of IL-33, and furthermore, mice treated with recombinant IL-33 exhibited reduced disease severity and improved survival (36). Similar to recombinant IL-25, mice treated with recombinant IL-33 had increased eosinophil infiltration into the colon and reduced epithelial damage. This protection by IL-33 was dependent on ILC2s, as the protection was abrogated in mice deficient for ILCs (*Rag2*^−/−^*γc*^−/−^) and adoptive transfer of ILC2s restored protection (36).

## REGULATORS OF PROTECTIVE AND PATHOGENIC CYTOKINES

IL-10 is a potent immunoregulatory cytokine that limits inflammation to commensal microbes and pathogens (103–105). IL-10 signaling in the intestine is critical for maintaining intestinal homeostasis and tolerance to the microbiota (106), as IL-10-deficient mice (*Il10*^−/−^) develop spontaneous colitis and are a widely used model for studying microbiota-driven intestinal immune dysregulation (107, 108). Kim et al. (109) demonstrated this association in a mouse model of *C. difficile* infection, where they observed impaired recovery from *C. difficile*-driven weight loss in *Il10^−/−^* mice compared to wild-type (WT) controls, which was accompanied by increased IFN-γ expression. However, Cribas et al. (110) found that *Il10^−/−^* mice were protected against acute *C. difficile* morbidity and mortality via elevated baseline IL-22 expression. This discrepancy in the protective vs detrimental effects of IL-10 in the two studies may be due to differences in intestinal inflammation at the time of *C. difficile* infection and the timing of IL-10 deficiency. The *Il10*^−/−^ mice in the study by Kim et al. (109) had no histological signs of inflammation prior to *C. difficile* infection, while Cribas et al. observed several protective IL-22-dependent immune response genes were elevated in the *Il10*^−/−^ mice prior to infection. (110). Blocking IL-10 signaling with monoclonal antibodies at the time of infection did not decrease the severity of *C. difficile* infection, while starting IL-10 blockade 3 weeks prior to infection improved host survival following infection as baseline IL-22 signaling increased in the prolonged absence of IL-10 (110). Abernathy-Close et al. (111) further showed that alterations to the intestinal microbiome driven by inflammation in *Il10*^−/−^ mice predispose them to *C. difficile* colonization in the absence of antibiotic treatment. Thus, loss of IL-10 signaling disrupts the microbiome to render the host susceptible to *C. difficile* infection but can simultaneously enhance baseline expression of protective defense cytokines. Prolonged loss of IL-10 signaling can drive an unchecked inflammatory response that impedes recovery following episodes of *C. difficile*.

IL-27 has both proinflammatory and immunoregulatory functions, where signaling on T cells leads to expression of T-bet and IFN-γ production while antagonizing Th17 cell development and inducing IL-10 production (112). IL-27 levels are elevated in blood and stool collected from *C. difficile* infection patients and infected mice (113). Mice deficient for IL-27 receptor (*Il27ra*^−/−^) had more severe disease suggesting a protective role for IL-27 in *C. difficile* infection (113). Mechanistically, IL-27 signaling regulated the inflammatory response, where *Il27ra*^−/−^ mice had elevated IL-6, IL-23, IL-17A, and CXCL1, and decreased IFN-γ and IL-10 compared to WT mice (113).

## LEPTIN RESISTANCE LINKS OBESITY TO INCREASED RISK OF *C. DIFFICILE* INFECTION

The adipocytokine leptin is a member of the IL-6 family of cytokines produced by adipose tissues (114). Leptin regulates appetite, and plasma leptin levels are elevated in obese individuals (115, 116). Furthermore, obesity is associated with increased risk of *C. difficile* infection in hospitalized patients (117, 118). In addition to regulating appetite and host metabolism, leptin regulates host inflammation, and a common human leptin receptor polymorphism (*LEPR* Q223R) increases the risk of infection, including *C. difficile* infection (68, 119–121). Mykoniatis et al. (122) first demonstrated a role for leptin signaling in *C. difficile,* reporting that leptin-deficient (ob/ob) and leptin receptor-deficient (db/db) mice lacked toxin A-induced intestinal inflammation. Leptin receptor signaling via the STAT3 pathway enhances chemokine expression and inflammatory cell recruitment during *C. difficile* infection (68). On the other hand, *C. difficile* infection patients homozygous for the inactive *LEPR* Q223R mutation (RR) have greater circulating neutrophils than *C. difficile* infection patients with QQ/QR genotype (65). *C. difficile-*infected mice with the same RR mutation also had more blood and cecal neutrophils and exhibited exaggerated inflammation and greater mortality than QQ genotype (65). The authors attributed this increase in mortality to increased expression of CXC chemokine receptor 2 on neutrophils from RR mice that was driven by dysbiosis of the intestinal microbiome in these mice (65). Thus, the increased risk of *C. difficile* infection with obesity may be driven by combined direct effects of leptin receptor signaling on the inflammatory response and indirect effects through the microbiome (114).

## EPITHELIAL REPAIR AND DISEASE TOLERANCE IN *C. DIFFICILE* INFECTION

When faced with an infection, the survival of the host has been typically thought to be dependent on the ability of the immune system to kill and eliminate the invading pathogen. A concept that is termed host “resistance” to pathogen invasion. A second defense strategy referred to as “disease tolerance” is also a vital component of the response to infections and is characterized by the ability to protect the host without negatively affecting the fitness of the pathogen (123–127). Indeed, many of the protective mechanisms of the cytokines described above were independent of reductions to *C. difficile* burden and its major toxins. The inflammatory response that is mounted to combat an infection is often accompanied by collateral immunopathology of host tissues. This is true with *C. difficile* infection as well, where the level of inflammatory markers in patients is correlated with worsened disease outcomes (17, 18). Thus, a productive immune response during *C. difficile* infection must balance controlling the pathogen and dissemination of pathobionts resulting from toxin-induced barrier damage while tolerating insult and restoring tissues damaged by the pathogen and inflammation.

Disease tolerance mechanisms during *C. difficile* infection include protection of intestinal epithelial cells from toxin-induced cell death and promoting repair of damaged intestinal barrier. Mileto et al. (128) demonstrated that *C. difficile* TcdB disrupts epithelial barrier integrity, exposing the stem cells within the crypt to toxin-mediated cell death and thus delaying repair processes. IL-22 stimulates intestinal stem cell proliferation and reduces epithelial cell loss from *C. difficile* infection (30). In other models of colitis and intestinal injury, IL-22 induces the expression of several anti-apoptotic (e.g., Bcl-2, Bcl-xL, Mcl-1, and Survivin) and mitogenic (e.g., c-Myc, cyclin D1, and CDK4) proteins to promote cell survival and facilitate tissue repair (129–131). Type 2 cytokines, including IL-4, IL-5, and IL-13, are also critical for wound repair typically following tissue damage by helminths. These cytokines recruit and activate eosinophils containing growth factors like TGF-β and matrix metalloproteinases that can facilitate wound healing and tissue remodeling (132, 133). Prostaglandin E2 plays an important role in protecting intestinal stem cells during DSS-induced colitis and irradiation injury (134, 135), and mice lacking genes for cyclooxygenase (COX)-1 or COX-2 have increased intestinal damage and greater clinical score compared to wild-type mice in DSS-induced colitis (136). In fact, a study by Zackular et al. (137) showed that treating mice with misoprostol, a synthetic prostaglandin, protected mice against severe disease and decreased intestinal permeability following *C. difficile* infection. Non-steroidal anti-inflammatory drugs (NSAIDs), which can inhibit COX enzymes and alter prostaglandin metabolism, are associated with increased risk of *C. difficile* infection in humans (138–141). In a mouse infection model, NSAID treatment worsened disease outcome from *C. difficile* infection, although this was independent of its effects on COX enzymes but instead caused mitochondrial damage in colonic epithelial cells, sensitizing these cells to intoxication (142).

Several other cytokines are associated with wound healing at epithelial barriers. Amphiregulin is a ligand of the epidermal growth factor receptor that facilitates epithelial regeneration and restores barrier integrity following infection or injury (143). Recombinant amphiregulin treatment reduced morbidity and mortality in a mouse model of *C. difficile* infection (144), suggesting this cytokine signaling pathway could be contributing to the host repair response to *C. difficile*-induced epithelial damage.

## CONCLUDING REMARKS

The immune response to *C. difficile* infection is orchestrated by a complex set of cytokines to promote both resistance and disease tolerance mechanisms. Among these, the consensus among published studies is that IFN-γ, IL-22, and type 2 cytokines (IL-4, IL-5, IL-25, and IL-33) protect against severe disease by facilitating bacterial control and promoting epithelial restoration processes, while IL-23 signaling is responsible for elevated neutrophil infiltration driving exacerbated inflammation and intestinal disease. The roles of IL-10 and IL-17A appear to be more nuanced and dependent on factors such as microbiome composition and cellular source. Future studies exploring the potential protective benefits of cytokine therapy in combination with existing treatments as well as methods for effective cytokine delivery will be valuable to develop a comprehensive treatment strategy to mitigate disease and accelerate recovery.
